# A spotlight on alkaloid nanoformulations for the treatment of lung cancer

**DOI:** 10.3389/fonc.2022.994155

**Published:** 2022-10-18

**Authors:** Sindhoor S. M., N. Raghavendra Naveen, GSN Koteswara Rao, Gopika Gopan, Hitesh Chopra, Moon Nyeo Park, Mohammed Merae Alshahrani, Jobin Jose, Talha Bin Emran, Bonglee Kim

**Affiliations:** ^1^ Department of Pharmaceutics, P.A. College of Pharmacy, Mangalore, Karnataka, India; ^2^ Department of Pharmaceutics, Sri Adichunchanagiri College of Pharmacy, Adichunchanagiri University, B. G. Nagar, Karnataka, India; ^3^ Department of Pharmacy, School of Medical and Allied Sciences, Galgotias University, Greater Noida, Uttar Pradesh, India; ^4^ Department of Pharmaceutics, NGSM Institute of Pharmaceutical Sciences, Nitte (Deemed to be University), Mangalore, Karnataka, India; ^5^ Chitkara College of Pharmacy, Chitkara University, Rajpura, Punjab, India; ^6^ Department of Korean Medicine, Kyung Hee University, Seoul, South Korea; ^7^ Department of Clinical Laboratory Sciences, Faculty of Applied Medical Sciences, Najran University, Najran, Saudi Arabia; ^8^ Department of Pharmacy, BGC Trust University Bangladesh, Chittagong, Bangladesh; ^9^ Department of Pharmacy, Faculty of Allied Health Sciences, Daffodil International University, Dhaka, Bangladesh

**Keywords:** nanoformulations, lung cancer, drug resistance, tumor targeting, alkaloids

## Abstract

Numerous naturally available phytochemicals have potential anti-cancer activities due to their vast structural diversity. Alkaloids have been extensively used in cancer treatment, especially lung cancers, among the plant-based compounds. However, their utilization is limited by their poor solubility, low bioavailability, and inadequacies such as lack of specificity to cancer cells and indiscriminate distribution in the tissues. Incorporating the alkaloids into nanoformulations can overcome the said limitations paving the way for effective delivery of the alkaloids to the site of action in sufficient concentrations, which is crucial in tumor targeting. Our review attempts to assess whether alkaloid nanoformulation can be an effective tool in lung cancer therapy. The mechanism of action of each alkaloid having potential is explored in great detail in the review. In general, Alkaloids suppress oncogenesis by modulating several signaling pathways involved in multiplication, cell cycle, and metastasis, making them significant component of many clinical anti-cancerous agents. The review also explores the future prospects of alkaloid nanoformulation in lung cancer. So, in conclusion, alkaloid based nanoformulation will emerge as a potential gamechanger in treating lung cancer in the near future.

## 1 Introduction

Lung cancers were the most extensively identified cancer and accounted for 18.4% of total cancer deaths worldwide, having a five-year survival rate of just 15% (1, 2). Based on histologic appearance and cell morphology, lung cancer can be categorized as small cell lung cancer (SCLC) and non-small cell lung cancer (NSCLC) (3, 4). NSCLC has further subdivided into adenocarcinoma, squamous and large cell carcinoma. Each of these NSCLC histological subtypes is unique and responds to various therapy (5). NSCLC accounts for 85% (6), and SCLC shares 15% of total lung cancers (1).

Tobacco smoking has been laid out as the primary source of lung cancers. This is mainly due to the exposure of the pulmonary systems to mutagenic chemicals found in inhaled smoke (7). Adenocarcinoma is the only kind of lung carcinoma that is not caused by smoking. It occurs due to exposure to carcinogenic substances such as asbestos, radon, and other types of radiation (8).

Typical symptoms of lung cancer include cough, hemoptysis, dyspnea, and systemic signs, including anorexia and loss of weight. An early lung cancer diagnosis is vital as failure will lead to progression to the advanced metastatic stage. Diagnosis techniques commonly involve chest radiography, computed tomography and possibly positron emission tomography (9).

Current treatment regimens for lung cancer mainly depend on the type of malignancy, stage at the time of diagnosis, and the patient’s physical condition. The primary therapy for non-metastatic lung malignancies is surgical removal/resection. However, this approach can only be employed in 10%–20% of patients and is restricted by the number and location of lesions. Also, surgeries may not altogether remove the tumor, especially in small cell lung cancer, where the tumor cells rapidly divide because of its metastatic properties (10, 11). In cases where the cancer is at an advanced stage, surgery may not be an option and chemotherapy is employed to prolong lives, reduce symptoms, and enhance patients’ quality of life with lung malignancies. Platinum-based medicines such as cisplatin and carboplatin are used in standard first-line chemotherapy regimens for lung cancer. On the other hand, Platinum-based chemotherapy has a broad range of dose-limiting side effects, including nephrotoxicity, anemia, intestinal damage, peripheral neuropathy etc. (12).

Radiation therapy is another commonly used treatment for lung cancer. It involves killing the tumor cell by the bombardment of high energy particles in the form of radiation. Radioactive isotopes like iodine are administered systemically to eradicate the cancer cell as part of the therapy. Radiotherapy is effective in damaging tumor cells; however, it also damages healthy lung cells. Other side effects associated with radiotherapy include nausea, emesis, bone marrow issues, hair loss, and exhaustion, among others, for which there are no preventative methods (13). The National Cancer Institute (USA) has encouraged research into the potential anti-cancer properties of plant extracts due to the adverse effects of existing therapy regimens (14, 15).

Herbal medicines have been used for ages to treat various disorders, including lung cancer. They have been proven effective in prolonging survival time, reducing the side effects of chemotherapy and enhancing the quality of life in lung cancer patients (16–18). The therapeutic activity of these plants is mainly due to the secondary metabolites, especially alkaloids (19). Alkaloids belong to a group of compounds with a ring structure and a nitrogen atom. A nitrogen atom is found inside a heterocyclic ring structure in most alkaloids. Due to their endless supply of systems, minimal toxicity, and good stability, plant-based alkaloids have been utilized to extract and synthesize hundreds of drugs used to treat various illnesses (20).

Recent investigations have shown that plant-based alkaloids have been influential in suppressing oncogenesis. The anti-cancer activity of the Phyto alkaloids is mainly attributed to Topoisomerase I inhibition and suppression of microtubule dynamics (21, 22). These compounds also can modulate the vital signaling pathways involved in the cell cycle, metastasis and proliferation, making them essential tools in the fight against lung cancer (23). However, most alkaloids extracted from plant sources have low solubility and permeability, resulting in poor bioavailability. Phytoalkaloids also lack selectivity towards cancer cells and undergo indiscriminate tissue distribution, leading to organ accumulation and toxicity. The development of resistance through multiple pathways is another hurdle in using these phytochemicals. Furthermore poor pharmacokinetic characteristics of these phytomedicine arising from rapid metabolism lead to inefficient delivery of the phytomedicine to the target site resulting in clinical failure (24–27).

Nanotechnology-based drug delivery systems have been envisaged to overcome drawbacks associated with traditional phytochemicals like alkaloids. The performance of these nanoformulations can be improved by modifying their composition, particle size and surface characteristics to increase their efficacy, reduce side effects and overcome drug resistance. It is expected that these phytochemicals incorporated into nanoformulations will have altered bioavailability, permeability and toxicity profiles and will have more efficacy in cancer therapy (28, 29).

Hence, this study attempts to review the recent updates on how alkaloid nanoformulations effectively manage lung cancer. In addition, we also highlight the different Phyto alkaloids that can be used for lung cancer therapy, along with their mechanism of action.

## 2 Alkaloids for lung cancer therapy

Natural resources are crucial origins of new leads, therapeutic drug molecules, and new chemical entities. Since the origin of phytomedicines, alkaloids have achieved increasing admiration due to their undeniable physiological attributes (30). In the frame of reference to evolution, several plants utilized these secondary metabolites for safeguarding them from herbivores (31). Later, they were soon used by ancient past hunters, who converted them to venom to poison arrows heads for hunting purposes (32). Subsequently, by the inception of the 19th era, various studies were conducted on these compounds. Over the years, their biological functions have become evident, got acknowledged in the medical disciplines, and turned them out as a significant constituent of various drugs (33, 34). More than 3,000 discrete type alkaloids were identified. Some alkaloids have also been found in animals. Usually, alkaloids are a distinctive class of compounds that comprises cyclic assembly supported by one basic nitrogen atom integrated within it. They are extensively distributed in the kingdom Plantae and occur mostly in plants about Leguminosae, Menispermaceae, Ranunculaceae, Loganiaceae, and Papaveraceae families. The exploration of plant-based drug molecules is intended to develop several anticancer agents through alkaloids. Investigation of several alkaloids from medicinal and herbal plants exhibited anti-proliferative and antineoplastic effects both *in vitro* and *in vivo* testings over extensive range of cancers. The electronic accessible databases were screened and summarized various isolated alkaloids for the antiproliferative effects in treating lung cancers. An overview of natural alkaloids with their sources and mechanism of action is given in **Table 1**.

**Table 1:** Mechanisms of action of some prominent alkaloids in the treatment of lung cancer.

Sl. No	Alkaloid	Sources	Mechanism of action
**Indole Alkaloids**			
1.	Vinca Alkaloids	*Catharanthus roseus* (Whole Plant)	Microtubule depolymerization and mitotic spindle destabilization ultimately lead to cell cycle arrest and cell death.
2.	Camptothecin	*Camptotheca acuminate* (Bark, Cortex, And Fruits)	Formation of Topoisomerase-Camptothecin complex, which inhibits topoisomerase I resulting in DNA damage and cell death
3.	Aplicyanins	*Aplidium cyaneum* (Antarctic tunicate)	Induction of apoptosis in cancer cells by activation of caspase. In addition, they also act by targeting the VEGF(Vascular endothelial growth factor) signal pathway and extracellular degradation of the matrix
**Isoquinoline alkaloids**			
4.	Sanguinarine	*Sanguinaria Canadensis* (Roots)	Suppression of cell growth and induction of apoptosis by downregulating the JAK/STAT pathway. Moreover, it induces apoptosis through the production of reactive oxygen species (ROS)
5.	Liriodenine	*Cananga odorata* (Barks)	Blocking of G2 to M phase transition of the cell cycle and induction of caspase activation and apoptosis in human lung cancer cells.
6.	Tetrandrine	*Stephania tetrandra* (Roots)	Suppress cell growth and induces apoptosis by inhibition of by activation of intercellular adhesion molecule-1 (ICAM-1)/hypoxia inducible factor (HIF)-1α HIF-1α/vascular endothelial growth factor (VEGF) pathways.
**Pyrrole and pyrrolizidine alkaloids**			
7.	Tambjamine	*Tambja eliora* *(Marine sources)*	Triggers imbalance in cellular homeostasis, causing dysfunction of mitochondria and deacidification of lysosomes resulting in necrotic cell death
**β carboline benzoquinolizidine alkaloids**			
8.	Oxymatrine	*Sophora flavescens* (Shrub)	Induces reduction in serum and glucocorticoid regulated kinase 3 (SGK3) by promoting the expression of miR-367-3p. It also causes cessation of the cell cycle at the G0/G1 phase and impedes the NF-kB signaling pathway.
9.	Punarnavine	*Boerhaavia diffusa* (Whole Plant)	Inhibits the expression of matrix metalloproteinase-2 and vascular endothelial growth factor(VEGF), resulting in cell death.
10.	Brucine	*Strychnos nux-vomica* (Seeds)	Induce apoptosis by blocking G0/G1 phase of the cell cycle through downregulation of Cyclin D1 and Cyclin E expression. In addition, they cause cell death by inhibition of COX2 expression and release of Prostaglandin E2 (PGE2)
11	Piperine	*Piper nigrum* and *Piper longum (fruits and roots)*	Triggers p53-mediated arrest of cell cycle in G2/M phase and apoptosis through the activation of caspase-3 and caspase-9 cascades

Similar to secondary metabolites, the usual chromatographic techniques extract and identify the alkaloids. Formerly paper and thin-layer chromatography were extensively used. Yet, nowadays, the high-performance liquid chromatography (HPLC) technique is mainly preferred to separate alkaloids (35). This is an exact method and has the potential to detect small amounts of a compound. Additional techniques for the isolation of the alkaloids include ultrasonic extraction, microwave-assisted extraction, supercritical CO_2_ extraction, and a combined method using ultrasound and surfactants.

### 2.1 Indole alkaloids

Indole alkaloids obtained from medicinal plants and herbs exhibited a cytotoxic effect on cancer cell lines. They exert influence on apoptotic proteins and thus result in autophagy, necroptosis, and apoptosis

#### 2.1.1 Vinca alkaloids

The compounds such as vinblastine, vincristine, and vindesine were obtained through species *C.roseus* of the Apocynaceae family and are broadly considered antitumor compounds. Alkaloids of these families cease cell growth by altering tubulin dynamics at the plus and minus end of spindle microtubules. Vinblastine and Vincristine are commonly employed in drug combinations and other chemotherapy drugs to manage different cancers such as leukemias, lung cancers, Kaposi sarcoma, and lymphomas (36, 37). Vinblastine, an autophagy maturation inhibitor, exhibited synergism when combined with nanoliposomal C6-ceramide (an agent that initiates autophagy). The combined effect increased cell death in human hepatocarcinoma (HepG2) and human colon (LS174T) cell lineages by increasing accumulation in autophagic vacuoles and minimizing their maturation.

The combination therapy repressed Beclin-1 protein knockout, which is involved with autophagy (38). Presently, semi-synthetic vinca alkaloid derivatives, namely vinorelbine, vindesine, vinfosiltine, and vinorelbine, have been launched. The derivatives are used solely or combined with other phytochemicals to combat various cancers (39). As per literature reports, nearly 64 cultivars of C. roseus were examined for vinca alkaloids where the maximum concentrations of serpentine were present in Cooler Rose Hot. Recently, an endophytic fungus isolated from C. roseus was recognized as an alternate procedure for producing different vinca alkaloids (40).

#### 2.1.2 Camptothecin

Camptothecin and its derivatives are quinoline alkaloids containing a pentacyclic structure. They are obtained from bark, cortex, and fruits of the plant Camptotheca acuminata and are known to exhibit anticancer effects (41). Camptothecin and its derivatives are produced by using various synthesis methods. The lactone group of camptothecin is very reactive to hydrolysis and yields carboxylic acid derivatives—hydrolysis results in depletion of camptothecin antitumor activity. The anticancer effect of this class is attributed to the formation of flexible strand breaks in DNA and interfering with the normal cell cycle. Camptothecin is believed to be bound with topoisomerase I and DNA, forming a ternary complex and thus hampers the reassembly of the single-chain DNA strands (42). At that instant, camptothecin interposes between the nucleobases in the DNA strands.

#### 2.1.3 Aplicyanins

A research study described six novel bromoindole alkaloids, namely aplicyanins A- F, obtained from tunicate *Aplidium cyaneum* by chloroform-methanol extraction. It was screened for cytotoxic potency over HT-29 colon cell lines, A-549 lung adenocarcinoma cell line, and breast (MDA-MB-231) cancer cells. Among them, aplicyanins B, D, E and F exhibited notable cytotoxic effects over the tested tumor cells (43).

#### 2.1.4 Mappianins

Mappianines A-E is monoterpene indole alkaloids obtained using the stem of *Mappianthus iodides.* All these compounds were studied for cytotoxic potency against various cancer cell lines such as lung (NCI-H460), liver (HepG2 and Bel-7404) *etc.* Amongst them, mappianine B exhibited modest cytotoxicity over different cancer cell cultures (34, 44).

### 2.2 Isoquinoline alkaloids

Many isoquinoline alkaloids exhibited antiproliferative effects by autophagy-dependent cell death interceded by the gene *ATG5*. In most studies, the up-regulated and down-regulated process of different proteins involved in the apoptosis pathway has also been investigated.

#### 2.2.1 Sanguinarine

Sanguinarine is a quaternary benzo phenanthridine alkaloid obtained from species *Sanguinaria canadensis* L. and *Chelidonium majus* L belonging to the Papaveraceae family (45). Sanguinarine is demonstrated to possess anticancer potentials (46) and is now gaining recognition by researchers. *In vitro* testing data revealed that this alkaloid exhibits antitumor effects at concentrations below 10 µmol in many cases. Sanguinarine ceases the cell cycle at different stages or may induce apoptosis in diversified cancer cells. Sanguinarine promoted cell death in pancreatic cancer cell lineages (Aspc-1 and Bxpc-3), lung cancer human cell lineages (A549) and was found effective to counter melanomas (47, 48).

#### 2.2.2 Liriodenine

Liriodenine, an alkaloid obtained from *Cananga odorata* of the Annonaceae family, displayed anti-proliferative, cytotoxicity, and pro-apoptotic effects in lung cancer cells of humans, and it was also noted to be a forbidding inhibitor of type II topoisomerase both *in vitro* and *in vivo* (49).

#### 2.2.3 Jorumycin

Jorumycin was obtained from pacific marine nudibranch *Jorunna funebris*, displayed cytotoxicity against various cell lines, i.e., lung (A-549), leukaemia (P-388), murine transformed cells (NIH3T3), and melanoma cells (MEL-28) (50).

#### 2.2.4 Tetrandrine

Tetrandrine is a bisbenzylisoquinoline group of alkaloids isolated from the roots of *Stephania tetrandra*. It has an extensive mixture of therapeutic properties like immunomodulation, anti-hepatofibrogenetic, anti-inflammatory, antiarrhythmic, antiporter hypertension, anti-tumor, and neuroprotection (51). Usually, it exhibits its anti-tumor effects in micromolar concentrations. Based on the tumor cell type, tetrandrine results in cell cycle arrest at various stages (52, 53) and also induce programmed cell death in different human cancers such as leukaemia, bladder, colon, hepatoma, and lung (54, 55). In addition, *in vivo* studies have illustrated the significance of tetrandrine against cancer activity. Tetrandrine co-administration may aid to reinstate the susceptibility of MDR cancer cells to doxorubicin, paclitaxel, docetaxel, and vincristine by suppression of P-glycoprotein.

### 2.3. Pyrrole and pyrrolizidine alkaloids

Several pyrrole alkaloids displayed significant anticancer effects through necrosis, programmed cell death and autophagy. Initiation of autophagy is interrelated with an expression of the Beclin-1 autophagic gene and a rise in the information of LC3B. Upon treatment with pyrrole alkaloids, necrosis is induced, and dysfunction of mitochondria occurs, leading to cell death.

#### 2.3.1 Tambjamine analogues

Tambjamine analogues exhibit cytotoxicity by disturbing homeostasis in cellular ions leading to mitochondrial malfunction and deacidification of lysosomes and causing tissue death (necrosis) in lung cancer cells (56).

#### 2.3.2 Dibromophakellstatin

Dibromophakellstatin is a tetracyclic brominated pyrrole-imidazole alkaloid obtained from the ocean sponge *Phakellia mauritiana*. It is cytotoxic to the human ovary (OVCAR-3), kidney (A-498), brain (SF-295), lung (H-460), and melanoma (SK-MEL-5) cell lines (57) Furthermore, it showed *in-vitro* cytotoxic effects against various cancers like ovarian (OVXF 899L), lung (LXF 529L), uterus (UXF 1138L), and glioblastoma (CNXF 498NL). Also, this study disclosed that pyrrole moiety debromination might result in deprivation of entire anticancer activity.

### 2.4 Phenanthroindolizidine alkaloids

This class of alkaloids comprises a phenanthrene ring mingled with the saturated indolizine ring. These alkaloids displayed prominent antitumor activity in counter to different cancer cell lines by modulation of apoptotic pathway (58). The noted antiproliferative activities were associated with arresting cell cycle regulation at various stages by forbidding the expressions of cell mediators.

#### 2.4.1 Hispiloscine

Hispiloscine is extracted from Ficus hispida Linn’s stem bark and leaves. It inhibited cell proliferation in breast (MDA-MB-231 and MCF-7), lung (A549 and MRC-5) and colon cancer (HCT-116) cell lines (59), and colon cancer (HCT-116) cell lines (34).

### 2.5 β carboline benzoquinolizidine alkaloids

β carboline benzoquinolizidine alkaloids include harmine, harmane, harmalol, harmaline, and tryptoline. Carboline alkaloids are obtained from *Peganum harmala* L. They were known to possess antitumor activity in human promyelocytic leukaemia, prostate carcinoma, and gastric cancer by elevating Phosphatase and Tensin Homolog (PTEN) levels and reducing extracellular-signal-regulated kinase. Carboline alkaloids target the NF-kB signaling pathway, and an additional course of action includes the generation of G-Quadruplexes. Carboline alkaloids from G-quadruplexes comprise tetra guanine nucleobases arranged in 3-D square planar geometry. This positioning shows the impact of the regulatory role on genes, particularly on oncogenes, hence serving as anticancer agents against human promyelocytic leukaemia, prostate carcinoma, and stomach cancers (60). The G-quadruplexes formation is a novel method in cancer therapy and possesses favorable applications in designing anticancer agents for inhibiting p-Glycoprotein, targeting ABCB1 and HER2 (61).

#### 2.5.1 Oxymatrine

Oxymatrine, obtained from the shrubs of Sophora flavescens Ait., was studied extensively because of its distinct molecular actions (62). Oxymatrine induced cytotoxicity concerning dose and time was studied in various cancers and carcinomas (63–65). These multiple targets are accompanied by different mechanisms that are more molecular by these alkaloids. Oxymatrine is capable of activating the intrinsic caspase pathway and persuading apoptosis. The hindmost is associated with the up-regulation of Bax and p53, the down-regulation of Bcl-2, cessation of the cell cycle at the G0/G1 phase, and impeding NF-kB signaling pathway. In addition, oxymatrine decreases the expression of many other genes and mechanistic pathways. Dysregulation of pathways brings on disturbance in cell fate specification, migration, and proliferation and thus promotes apoptosis of cancer cells.

#### 2.5.2 Punarnavine

Punarnavine, an alkaloid derived from the *Boerhaavia diffusa* Linn plant, inhibited the growth of metastatic melanoma cells (B16F-10) in mice. In the lung tissue of animals with metastases, it also inhibits the expression of matrix metalloproteinase-2 (MMP-2), MMP-9, ERK-1, ERK-2, and vascular endothelial growth factor (VEGF) (66).

#### 2.5.3 Brucine

Brucine is a potent alkaloid isolated from the seeds of *Strychnos nux-vomica* L., showed anti-proliferative effects in human colon and lung cancer cells and can cease the cell cycle at the G0/G1 stage (67). Thus, *in vivo* and clinical trials of these new apparent alkaloids mentioned above, namely noscapine, liriodenine, isogravacridone, clausenidin, cycleanine, cryptolepine, and brucine, can halt the cell cycle and furnish us with new antitumor agents with potential activity and minimal toxicities. Further, Alkaloids can also be evaluated for synergic effects since they represent the desire for taxol, vincristine, and vinflunine’s anticancer actions. They interfere with the rapid growth of the human lung tumor cell lines PC-9 by ceasing cell cycle regulation at G0/G1 stage by decreasing cyclin E mRNA activation and protein expressions of D1 and E cyclins (68).

#### 2.5.4 Piperine

**Figure 1:**
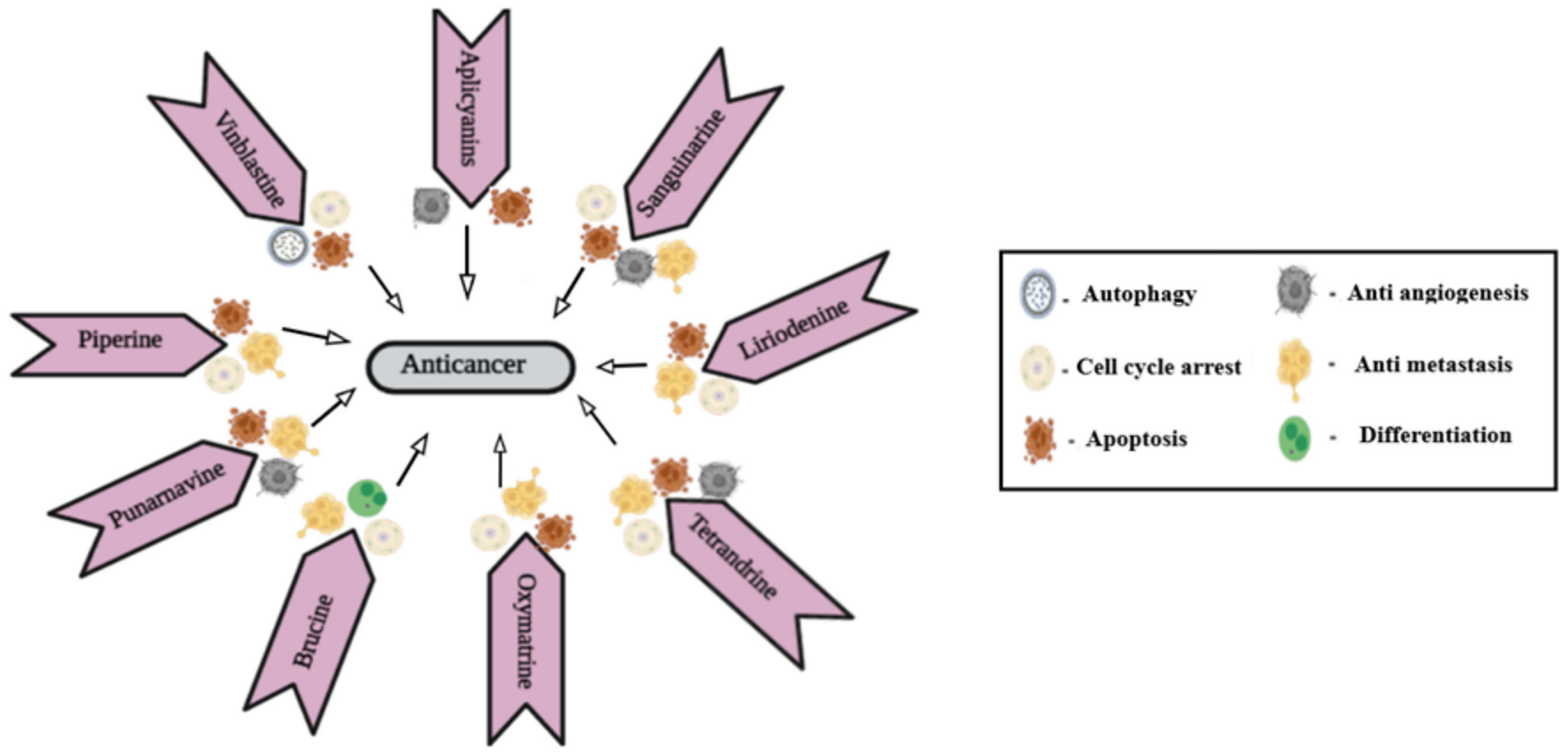
Mechanism of action of different alkaloids.

Piperine is an N-acylpiperidine alkaloid obtained from *Piper nigrum* and *Piper longum*. A dose of 200 _M/kg piperine is effective against metastatic lung cancer caused by B16F-10 melanoma cells in mice; it repressed phorbol-12-myristate-13acetate (PMA), which induced invasion of tumor cells. Piperine inhibits c-Fos- cAMP- response element (CRE) regulation, NF-kB and activated transcription factor 2 (ATF-2). It represses PMA-induced MMP-9 activation by inhibiting protein kinase C extracellular signal-regulated kinase (ERK) 12 and dwindling NF-kB/AP-1 activation (69). It also suppresses P-glycoprotein (P-gp) and cytochrome (CYP3A4) activity, which interferes with cell metabolism and also re-sensitizes multidrug-resistant (MDR) tumor cells (70). Mechanism of action of different alkaloids is given in **Figure 1**.

The molecular action and signaling pathway regulation through which all the above alkaloids exhibit their anti-neoplastic activity is summarized in the below **Table 2**.

**Table 2:** Molecular action and signaling pathway regulation of alkaloids.

S. No	Alkaloids	Signaling pathway regulation	References
1.	Indole alkaloids	Inhibit JAK/STAT signaling pathway and Akt/NF-кB signaling pathway	(71)
2.	Isoquinoline alkaloids	Inhibit JAK/STAT signaling pathway	(72)
3.	Benzoquinolizidine alkaloids	Inhibit JAK/STAT signaling pathway	(72)
4.	Pyrrolizidine alkaloids	Suppress the p53 signaling pathway	(73)
5.	Phenanthroindolizidine Alkaloids	Inhibition of NF-κB signaling pathway,	(74)
6.	Benzoquinolizidine alkaloids	Dephosphorylation of PI3K and Akt in the PI3K/Akt signaling pathway	(75)

Despite the potential activity of plant cancer against lung cancer, their effectiveness is limited by their low bioavailability and non-selectivity. Selective targeting is crucial in lung cancer therapy, where the phyto alkaloid can be specifically targeted to cancer cells without affecting the normal cells. Nanoparticle-mediated therapies, on account of nano-sized particles, can pass through the leaky tumor vasculature, resulting in passive targeting *via* the EPR effect. Conjugated nanocarriers containing the phytoalkaloids that bind to over-expressed cells can be effectively used for active targeting in lung cancer therapy. Moreover, the biodistribution of Phytonanocarriers can be improved by altering their surface characteristics based on the target of interest in lung cancer therapy. Thus to ensure selectivity in therapy, loading the alkaloids into nanotechnology-based systems becomes necessary. Besides that, nanocarriers have high surface-area-to-volume ratios, enabling better loading of the phytoconsituents (76, 77). The nanocarriers containing alkaloids that have been investigated for their potential in lung cancer therapy is discussed in detail in ensuing sections.

## 3 Different types of nanoformulation containing alkaloids for management of lung cancer

### 3.1 Organic nanoparticles

- Lipid-based nanoparticles or liposomes
- Polymer-based nanoparticles
- Polymer micelles
- Dendrimers

Nowadays, nanoparticles are the widely used carrier systems in the management of lung cancer. These are prepared from lipids, polymers (natural/semisynthetic/synthetic), and metals. These nanoparticles are highly used for cancer research both *in vivo* and *in vitro*. Nanoparticles have the potential for local drug delivery and sustained release (78). It showed improved bioavailability through prolonged circulation (79). The properties of nanoparticles can also be tailored through surface modification with certain ligands to achieve the targeting of specific tissues like cancer cells (Eliseo 80).

#### 3.1.1 Lipid-based nanoparticles (or) liposomes

Liposomes are nanoscaled spherical vesicles that can hold hydrophobic drugs in their bilayer. In contrast, the aqueous core can keep the hydrophilic medicines, and their exterior appearance is similar to that of a biological membrane (81). Liposomes have various distinguishing characteristics, like physical stability, non-toxicity, high vascular density, external stimuli responsivity, and targeted drug delivery (82). The most common phospholipids are phosphatidylethanolamine and phosphatidylcholine (83, 84).

Targeted techniques such as active/passive, pH/magnetic/thermo/stimuli-responsive targeting deliver therapeutic compounds using synthesized liposomes. Compared to drug solutions, such selective targeting enhances pharmacodynamic and pharmacokinetic profiles, modulates therapeutic agent release, and decreases toxicity (85, 86). This strategy boosts therapeutic drug bioavailability at the target location, reduces adverse effects, and improves overall therapeutic response (87). “Intelligent liposomes” are employed as an efficient drug carrier for the treatment of lung cancer (88). These liposomes are also known as “smart liposomes”, as they perform better than conventional liposomes due to surface modifiers. These “smart liposomes” can offer accurate targeting of cancer cells and show minimum risk towards multi-drug resistance (89).

Liposomes offer appreciable loading of a variety of drugs and reduction of systemic toxicity, thereby enhancing the overall stability (90). Several liposome-based alkaloid anticancer drugs are in different stages of clinical trials for minor cell lung cancer treatment. They include irinotecan and lurtotecan liposomes (91).

Wijagkanalan et al. used an intratracheal injection of mannosylated liposomes to assess the targeting efficacy of alveolar macrophages (Man-liposomes) (92). Mannose receptors, belonging to the lectin family (C-type), are available on the alveolar macrophage surface and have a high affinity for mannose terminal molecules (B L 93). Moreira et al. investigated that the growth factor antagonist G has preferred sterically stabilized PEGylated liposomes in H69 cell lines of human classical small-cell lung cancer (94). This antagonist G coupled liposomes showed increased nuclear transport of loaded doxorubicin to target cells, boosting the drug’s therapeutic activity compared to conventional liposomes. The radiolabelled (125I tyraminylinulin) liposomes showed a long half-life of 13 hours in mice.

Folate receptors, typically overexpressed on the epithelium of tumor cells, are utilized for targeted drug delivery through surface-modified liposomes. Lee and Low used polyethylene glycol (PEG) to create folate-conjugated liposomes that were then loaded with doxorubicin (95). In KB (human nasopharyngeal epidermal carcinoma) cells, the absorption of folate-PEG-liposomal doxorubicin was 45 times more than non-targeted carriers and 1.6 times higher than free doxorubicin, with 86 and 2.7 times higher cytotoxicity, respectively.

Because of their unique characteristics, lipid-based nanocarriers were found as attractive drug delivery systems of anticancer agents (96, 97). However, conventional liposomes suffer from limitations like low systemic stability and rapid degradation through opsonization by the reticuloendothelial system (80, 98).

To overcome such limitations, the liposomes were surface modified by the researchers to tailor the properties and combat the existing limits. The surface treatment with a hydrophilic polymer like polyethene glycol confers stability to the liposomes’ surface. It offers protection from opsonization, thereby escaping from the reticuloendothelial system showing increased circulation time in the blood (99). Long-circulating liposomes of a different variety, known as stealth liposomes, have lately gained much attention in treating lung malignancies. It increases the delivery rate and interaction rate at the targeted tumor region since it has a more extended residence and circulation period (100).

PEG grafter liposomes are promising in anticancer therapy (101). Several chemotherapeutic drugs, including doxorubicin and vincristine, have shown promising results during *in vitro* and *in vivo* preclinical studies.

In lung cancer management, lipid-based nanoparticles are advancing as a potential delivery mechanism for medicines and genes. For the treatment of NSCLC, cisplatin is the drug of choice. Furthermore, only three platinates have been effectively utilized in clinics: cisplatin, carboplatin, and oxaliplatin (102)

On the other hand, Cisplatin has been linked to the cause of nephrotoxicity in 20% of patients taking high doses (12). As a remedy to minimize the toxicity, cisplatin has been encapsulated in liposomes (lipoplatin) and used for NSCLC and pancreatic cancer with improved therapeutic efficacy (103).

A randomized Phase III research on Lipoplatin for non-squamous NSCLC revealed exciting and promising results. Lipoplatin with paclitaxel was utilized as first-line therapy for non-squamous NSCLC in this trial, and responses were compared to those receiving cisplatin plus paclitaxel. The Lipoplatin arm had shown a 59.22% tumor response rate compared to 42.42% of the cisplatin arm, which was statistically significant.

Paclitaxel, one of the promising anticancer drugs, suffers from low solubility. Even though the problem has been addressed by intravenous administration using solubilizers like Cremophor RL and polysorbate 80, researchers still tried to overcome the unsolicited responses like myelosuppression and peripheral neuropathy, or hypersensitivity (104). Hence, liposomal paclitaxel was developed to address these side effects and evaluated for improved efficacy. Kaudelka and Turanek have developed liposomal paclitaxel formulations with a significantly increased tolerated dose than classical formulations (105). There are several studies evaluating liposomal paclitaxel for lung cancer treatment. Wang et al. have shown promising results of paclitaxel liposome on infusion into NSCLC patients in phase I clinical trials concerning toxicity (106).

Further studies on paclitaxel liposomes are in progress to validate their efficacy and safety. Liposomes were also tested to enhance therapeutic effectiveness with reduced side effects of drugs like doxorubicin and a novel SN-38 molecule (107, 108). To verify the suitability as a first-line treatment of advanced NSCLC, comparative studies are in progress towards combination therapy of paclitaxel micelles with cisplatin against paclitaxel injection made of Cremophor EL (Eliseo 80).

As per clinical investigations, polymeric micelles are known to lessen adverse therapeutic effects and successfully against a variety of refractory malignancies, including triple-negative breast cancers and lung cancer (109), demonstrating their clinical promise. Another potentially helpful characteristic of nanocarriers for subcellular medication targeting has recently gotten much attention (107).

Nanomedicine’s subcellular drug targeting might increase the pharmacological action of loaded medications by improving subcellular drug distribution (110). Avoidance from drug efflux pump is possible by releasing drugs in acidic organelles like lysosome and endosome using nanocarrier systems (111, 112).

Intracellular activation of the loaded drug cisplatin has been developed using a nanoparticle carrier system following a structure-activity relationship. A new lipid-based nanoplatinate was created using this approach, making the cisplatin release a pH-dependent process. A significant improvement in the anticancer efficacy has been reported in tumor development delay and decreased systemically and nephrotoxicity in breast and lung malignancies (113).

Several nanoparticle-based therapeutic techniques are now in pre-clinical and clinical trials, awaiting Food and Drug Administration (FDA) or European Medicines Agency (EMA) approval (114). Various nanoparticle-based formulations were authorized to treat multiple tumors following clinical studies. The first FDA-approved cancer nanomedicine was Abraxane, which used liposome-based nanocarriers to encapsulate Doxil (an anti-tumor medication). After then, other nanomedicines, including DaunoXome, Marqibo, and Myocet, were authorized (115).

A lipid-based nanoparticle, DOTAP/Chol TUSC2, is now undergoing phase I clinical research to see if it will help patients with metastatic lung cancer. A solid lipid nanoparticle carrier p53 has shown better efficacy in treating transfected p53-null H1299 lung cancer cells than a commercially available product, Lipofectin (116). Furthermore, “GenexolPM”, a polymeric nanoformulation proposed for NSCLC treatment, is now under phase II clinical trials (117).

Following the commercial and clinical success of many NDDS formulations, significant efforts are being made to maximize the therapeutic potential of licensed nanomedicines. We’ve compiled a list of the most recent clinical trials for alkaloid based lung cancer formulations (**Table 3**).

**Table 3:** Summary of clinical trials on alkaloid based formulation for lung cancer.

Rank	Title	Conditions	Interventions
1	GEfitinib Plus viNOrelbine in Advanced EGFR Mutated NSCLC. GENOA Trial	Non-Small Cell Lung Cancer	Drug: oral vinorelbine|Drug: Gefitinib
2	Effectiveness of an Enhanced Tobacco Intervention Protocol Compared to Standard Treatment in Helping Head and Neck and Lung Cancer Patients Starting Treatment to Reduce Cigarette Use	Lung Non-Small Cell Carcinoma|Head and Neck Squamous Cell Carcinoma	Drug: Nicotine Replacement|Drug: Bupropion Hydrochloride Controlled-release|Drug: Varenicline|Other: Tobacco Cessation Counseling|Other: Questionnaire Administration|Other: Quality of Life Assessment|Other: Best Practice
3	Phase 2 Study of EC145 Alone Versus EC145+Docetaxel Versus Docetaxel Alone in Participants With FR(++) 2nd Line Non-Small Cell Lung Cancer	Non-Small Cell Lung Cancer	Drug: EC145|Drug: EC145 + Docetaxel|Drug: Docetaxel|Drug: EC20
4	Study of Pembrolizumab, Lenvatinib and Chemotherapy Combination in First Line Extensive-stage Small Cell Lung Cancer	Small Cell Lung Cancer Extensive Stage	Drug: Lenvatinib|Drug: Pembrolizumab|Drug: Etoposide|Drug: Carboplatin
5	Vinorelbine in Mesothelioma	Mesothelioma	Drug: Vinorelbine|Other: Active Symptom Control
6	PembROlizuMab Immunotherapy Versus Standard Chemotherapy for Advanced prE-treated Malignant Pleural Mesothelioma	Pleural Mesothelioma Malignant Advanced	Drug: Pembrolizumab|Drug: Gemcitabine|Drug: Vinorelbine

#### 3.1.2 Polymer-based nanoparticles

Polymeric nanoparticles address significant limitations of the anticancer treatment and their drug delivery process (118). Polymeric nanoparticles offer encapsulation of high concentration of hydrophobic drugs, prolonged circulation of carriers, targeted drug delivery and improved therapeutic efficacy of anticancer drugs (119).

Abraxane, FDA approved albumin-bound paclitaxel nanoparticle, is a blockbuster for treating NSCLC and metastatic breast cancer (120). Jung et al. has stated the improved *in vitro* chemoradiotherapeutic efficacy of taxanes loaded polymeric nanoparticles in NSCLC using human lung cancer cell lines, A549 (121).

Hu et al. studied the effectiveness of polycaprolactone based nanoparticles loaded with paclitaxel alongside chrono-modulated chemotherapy and reported promising results (122). Further, in another study, mesenchymal stem cells were used to check the improved drug delivery of paclitaxel loaded nanoparticles (123; Eliseo 80).

Because of their ability to get tailored in terms of composition and form, polymeric nanoparticles have emerged as a viable carrier system for treating tumors in the field of nanotechnology (124).

Natural polymers like alginic acid, albumin, chitosan, gelatin, polypeptides and synthetic polymers like polycaprolactone, polylactide-co-glycolide, and polylactic acid are some of the polymers used in the development of polymer-based nanoparticles to treat cancer (80, 125).

However, adding a sulphide link to these polymeric nanoparticles modulates the release of the therapeutic medication (126, 127).

Polymeric nanoparticles coated with hyaluronan/polyethyleneimine were developed for the targeted release of docetaxel towards CD4 receptors of lung carcinoma cells (128). Jiang et al. studies the efficiency of a polylactide-tocopheryl PEG (1000) succinate-based nanocarrier system for delivering crizotinib to lung cancer patients (129).

Nanotechnology advancements will aid in the development of innovative cationic polymers to mute the siRNA genes in lung cancer. An *in vivo* study revealed that cationic polymers indicated 50% of targeted gene expression silencing. Another research found that low-molecular-weight polymeric nanoparticles may be used to silence the expression of several genes in endothelial cells (130).

The importance of aerosol drug delivery in lung cancer with the potential of reduced systemic toxicity has been reported (131). Improved anticancer activity of cisplatin has been reported with lung administration of gelatin-based nanoparticles in A549 lung adenocarcinoma cells (132). Xi et al. reported increased lung concentration of the drug upon pulmonary delivery of hyaluronic-cisplatin conjugate nanoparticles compared to intravenous cisplatin after 24 hours. They have also reported a low tissue/plasma ratio in both kidneys and the central nervous system with reduced dose-limiting toxicities (80, 133).

#### 3.1.3 Polymeric micelles

Lipid-based polymeric micelles are nanoparticles where the hydrophobic core can encapsulate the drugs with a hydrophilic shell. The circulation time of these particles is longer than other nanoparticles, thereby offering accumulation in solid tumors after administration (109). Kim et al., reported a novel polymeric micelle (Cremophor-free) formulation of paclitaxel which was approved for treatment of advanced NSCLC in South Korea and other European countries (134). Li has studied and reported the aggregation-induced emission based cisplatin loaded polymeric micelles for cellular imaging and chemotherapy (135). Decreased toxicity has been reported with paclitaxel and itraconazole encapsulated polymeric micelles in the treatment of NSCLC (136). Docetaxel loaded polymeric micelles modified with alpha-conotoxin were studied for targeted delivery to the A549 NSCLC cell lines (137).

Reshma et al. studied paclitaxel loaded galactoxyloglucan nanoparticles in lung cancer cells and reported them as a remedy for drug resistance. They have downregulated the expression of some multi-drug resistant proteins (138). These systems are also employed for the administration of cancer and ophthalmic medications. The toxicity of polymeric micelles encapsulated with paclitaxel and itraconazole was significantly reduced in NSCLC (136). In the A549 NSCLC cell lines, alpha-Conotoxin coated polymeric micelles loaded with docetaxel have targeted drug delivery to the 7-nAChR gene.

#### 3.1.4 Dendrimers

Dendrimers are highly ordered, repeatedly branched synthetic polymeric symmetrical nanosized molecules. They are covered with anionic, neutral, or cationic functional groups rendering great scope for drug delivery. These are globular in shape with monodisperse and homogenous characteristics (80, 139–141).

Dendrimers are made chemically by a controlled polymeric process that involves electrostatic and hydrophobic interactions. Dendrimers can be surface treated to enhance biodegradability (142). Because of their symmetrical structure, high payload, biocompatibility, and biodegradability, these nanocarriers play a promising role in cancer therapy (143). They also offer several conjugation points to demonstrate surface modification.

Dendrimers are considered a solution for solving the problems of drug candidates with poor solubility, toxicity or stability and have become promising carriers for enhancing their clinical applications (144).

The ability of dendrimers to use specific ligands against a target tissue and provide targeted drug delivery with enhanced therapeutic value stood as a significant advantage in choosing them as carriers in lung cancer (145). Several drugs indicated in lung cancer are coupled with dendrimers for enhanced therapeutic use with reduced toxicities. Doxorubicin was loaded into fifth generation PEGylated poly(amidoamine) dendrimers which resulted in increased therapeutic efficacy as well as specificity against lung cancer through its pH responsive characteristic (146, 147). PEGylated dendrimers (fifth generation poly(amidoamine)) are also used for improved aqueous solubility and targeted drug release of imatinib in cancer cells (148, 149).

Cisplatin being a popular anticancer drug, researchers attempted for its targeted drug delivery for reducing the side effects at healthy tissues and improving therapeutic benefits. Dendrimers are made target specific against folate receptor which are highly expressive in cancer cells. Hence, poly(amidoamine) dendrimers conjugated with folic acid are developed for the co-administration of cisplatin, human receptor R and siRNA towards the treatment of lung cancer. The results indicated that the *in vitro* chemo-biologic chemotherapy has shown promising therapeutic efficacy with reduced normal cell cytotoxicity in NSCLC cell lines and normal lung fibroblast (150).

### 3.2 Hybrid nanoparticles

- Solid lipid nanoparticles
- Nanostructured lipid carriers
- Lipid polymer based hybrid nanoparticles
- Nanosuspensions
- Nanoemulsions

### 3.3 Hybrid nanoparticles

Organic and inorganic nanoparticles have proved their efficiency in the treatment of cancer with several advancements in their fabrication. To have the added advantage of different substrates used for the development of nanoparticles, researchers have introduced hybrid nanoparticles which include solid lipid nanoparticles, nanostructured lipid carriers, lipid polymer based systems, liposome-silica hybrid nanoparticles, nanosuspensions, nanoemulsions etc. (151). Under this category, promising drug delivery platforms were reported for the management of pancreatic cancer (152, 153), breast cancer (154, 155), and metastatic prostate cancer (156). The biocompatible nature of lipids are merged with the structural integrity of polymer in addition to the capabilities of loading both hydrophilic and hydrophobic drugs (157, 158).

Even the coating of nanoparticles with naturally derived cell membranes (obtained either from red blood cells or leukocytes or platelets or cancer cells) could enhance the safety and potency relative to conventional nanoparticles (159, 160). The clearance from phagocytes can be minimized with such hybrid nanoparticles and the circulation time can be increased. A dual biomaterial coated nanoparticles like erythrocyte-platelet or erythrocyte-cancer cell hybrid nanoparticles were also shown to be performing well in cancer treatment with improved stability and enhance duration of circulation time (161–163).

#### 3.3.1 Solid lipid nanoparticles

Solid lipid nanoparticles, submicron colloid carriers (particle size range of 50 to 1000 nm), were studied well for their drug delivery functions, and the same has been attempted and found promising for treatment of lung cancer (164). Solid lipid nanoparticles are prepared by using either natural or synthetic lipids e.g., triglycerides, carnauba wax, cetyl alcohol, emulsifying wax, beeswax, cholesterol, and cholesterol butyrate (165–168). Solid lipid nanoparticles were reported for delivery of doxorubicin, paclitaxel, idarubicin, etoposide and camptothecins as anticancer agents (169).

Solid lipid nanoparticles have ability to encapsulate both hydrophilic and hydrophobic drugs and offer greater stability, longer circulation, and biocompatibility. Solid lipid nanoparticles were reported for their application in diagnosis of lung diseases and also for drug delivery to lungs (170, 171). For the last 3 decades, solid lipid nanoparticles have been explored by researchers to solve several drug delivery obstacles and hence found as potential drug carriers for anticancer agents too. They can provide improved bioavailability and sustained drug delivery of anticancer drugs. The suitability of solid lipid nanoparticles for lung cancer therapy is because of their safety profile attributed to their biocompatible lipids used in the fabrication (172). They offer high biocompatibility, low toxicity, improved drug targeting and ease of fabrication at low cost (173, 174).

Ligand-based surface alteration of solid lipid nanoparticles could enhance the target efficiency and allow them as suitable carrier systems for targeted drug delivery (175).

Reinstating the p53 gene because of its significant role in the effective induction of apoptosis has been successfully reported as a better therapy in cancer. Cationic solid lipid nanoparticles are used to transfect lung cancer cells with the p53 gene (116, 151).

Solid lipid nanoparticles were reported to show the enhanced antitumor activity of berberine alkaloids in NSCLC (176). Berberine hydrochloride, an isoquinoline alkaloid, has proved anticancer efficacy. Berberine shows an antitumor effect by inhibiting the proliferation of cancer cells. Berberine is also known to induce cell cycle at G1/G0 phase and apoptosis in cancer cells (177, 178).

However, poor aqueous solubility has limited its clinical application and further product development. Other limitations of berberine include low oral absorption and rapid metabolism (179, 180). Berberine loaded solid lipid nanoparticles were developed by Wang et al. for efficient encapsulation and sustained release of the drug molecule (176). They have investigated the drug release kinetics and the *in vitro* antitumor efficacy on several cancer cells (MCF-7, HepG 2, A549 & MCF-10A). The results indicated berberine solid lipid nanoparticles’ stability and narrow particle size range with a mean zeta potential value of -28.67 ± 0.71 mV. They have shown 70.33 ± 1.53% entrapment efficiency and 2.85 ± 0.04% drug loading efficiency. The *in vitro* drug release studies revealed the ability of solid lipid nanoparticles to sustain the drug release for up to 48 hours.

Further studies like cellular uptake, clone formation, cell apoptosis, and cell cycle arrest have powerfully demonstrated the enhanced antitumor efficacy of berberine on cancer cell lines, MCF-7 (176).

Wang and his team have showcased the evidence that solid lipid nanoparticles played a promising role in improving the antitumor efficacy of berberine. These systems are stable, safe and practically approachable systems for cancer therapy (176).

An alkaloid anticancer drug, paclitaxel has been used extensively to manage NSCLC, breast cancer and ovarian cancer for the last 3-4 decades (181–183). Hence, paclitaxel administration to the lungs has been attempted by researchers for better therapeutic efficacy.

Videira et al. formulated solid lipid nanoparticles loaded with paclitaxel by using the melt homogenization technique, where glycerol palmitostearate and polysorbate 80 are used as ingredients (184). Therapeutic efficacy was studied using MXT-B2 cancer lines from the lungs. B6D2F1 female mice were used to inoculate MXT-B2 cancer cells that developed experimental lung metastases. Paclitaxel loaded solid lipid nanoparticles through inhalation treatment have shown an efficient reduction of lung metastases number and size compared to the IV treatment with paclitaxel in B6D2FI female mice MXT-B2 cell model (185). The researchers claimed that the pulmonary administration of solid lipid nanoparticles loaded with paclitaxel had offered targeted drug delivery (intracellular drug targeting and delivery into the cytosol) that reduces the deposition of the drug at nontarget tissues, thereby minimizing the toxicity and increasing the therapeutic index of the drug (185).

Inhalable epirubicin and doxorubicin solid lipid nanoparticles were also reported with improved therapeutic advantages (152, 186). In another recent study, Yang et al. reported the efficacy of dry powder polylactide co-glycolide porous microspheres for coadministration of afatinib-loaded solid lipid nanoparticles and paclitaxel. They have shown improved bioavailability with sustained drug levels in lung tissues than in other tissues (187).

#### 3.3.2 Nanostructured lipid carriers

The major drawback of solid lipid nanoparticles includes the limited entrapment efficiency of the drug due to its highly organized crystalline structure of solid lipids and hence shows drug expulsion during storage (91, 188). As a remedy, researchers have introduced nanostructure lipid carriers as the next version of solid lipid nanoparticles (189). These can be considered modified versions of solid lipid nanoparticles where the liquid lipids are incorporated with solid lipid. Nanostructured lipid carriers have shown relatively improved drug loading capacity, due to their crystal imperfections and fatty acid chains (190, 191).

Compared to the drug solution, celecoxib encapsulated nanostructured lipid carriers have shown better deposition in lung tissues. The lung residence time was also increased with these carriers (91, 192).

Improved gene and paclitaxel delivery to the cancer cells was reported with transferrin linked nanostructured lipid carriers. *In vitro* and *in vivo* studies conducted in NSCLC cell lines of humans showed low cytotoxicity, high antitumor activity, and increased gene transfection (193, 194).

#### 3.3.3 Lipid polymer hybrid nanoparticles

Biocompatibility, biodegradability and integrity of polymeric nanoparticles are combined with the biomimetic characteristics of the liposomes to obtain the so-called lipid-polymer hybrid nanoparticles (195–197). With lipid polymer hybrid nanoparticles, the properties like size, surface charge, encapsulation efficiency, drug release and functionality can be tailored suitably to target a specific tissue (198).

Mandal et al. developed and evaluated the core-shell lipid polymer hybrid nanoparticles for the efficient delivery of erlotinib to treat NSCLC. Biodegradable polycaprolactone was used as a core polymer. In contrast, the lipid shell is comprised of hydrogenated soy phosphatidylcholine (HSPC) and 1,2-distearoylsnglycero-3-phosphoethanolamine-N [methoxy(polyethene glycol)-2000 (DSPE-PEG2000) to deliver erlotinib for lung cancer treatment. The results indicated a remarkable decrease in the proliferation of A549 cells after 72 hours, and the reason attributed is the high uptake of erlotinib loaded carriers by lung cancer cells. Hence these systems can be considered suitable for an optimal supply of anticancer drugs to lung cancer cells (199).

#### 3.3.4 Nanosuspensions

Even though not much is explored like other nano systems for cancer treatment, nanosuspensions still exist in the research arena. BIND-014, injectable suspension of docetaxel nanoparticles, is under phase-2 clinical trials as a second-line therapy for NSCLC (NCT01792479) (192). Qiao et al. formulated isoliquiritigenin nanosuspensions and found enhanced cytostatic effects in lung cancer cell lines (200). Huang et al. prepared and evaluated celastrol nanosuspensions for high drug loading and anticancer effect in breast cancer (201). The results showed improved bioavailability and stability with significantly enhanced tumor inhibition in comparison with celastrol suspension. Hence, nanosuspensions could be an effective and promising carrier system for lung cancer treatment.

#### 3.3.5 Nanoemulsions

Nanoemulsions are studied to improve the efficacy of nutraceuticals and pharmaceuticals (202). Some oils play multiple roles like oil phase, stabilizer, antimicrobial agents, and antitumor agents through their synergistic properties (203–205).

Jing Zhao et al. has reported the synergistic anti-tumor effect of a woody plant seed oil, namely fructus bruceae oil, and its oil phase and stabilizer roles. A poorly soluble natural alkaloid, evodiamine, has been loaded into the fructus bruceae oil-based emulsive nanosystems and evaluated for efficiency (Zhao et al.). The researchers found that the fructus bruceae oil nanoemulsion based evodiamine has shown antitumor activity in NSCLC A549 cells, whereas the free evodiamine doesn’t. This might be because of the increased uptake of the lung cancer cells’ alkaloids. The study proved that the nanoemulsion system has efficiently delivered the natural alkaloid.

### 3.4 Miscellaneous nanoparticles

#### 3.4.1 Metal nanoparticles

The nanoparticles based on metals and their derivatives are widely explored for their anti-cancer activity due to their ability to elicit drastically different responses in normal and cancerous cells. Metal nanoparticles are metallic particles with sizes ranging from 1-100nm. They include nanoparticles like silver (Ag), gold (Au), nickel (Ni), silica (SiO2), iron oxide, platinum (Pt), zinc oxide (ZnO), and titanium dioxide (TiO2). These metallic nanoparticles play a crucial role in cancer therapeutics due to their superior gene silencing, tumor targeting and effective delivery. Many of the phytochemicals or plant-based constituents like alkaloids have been conjugated with metal nanoparticles to prevent the degradation of the phytoconstituent from the cell’s external environment. Moreover, these metal nanoparticles improve the passage of the phyto compounds through the biological membrane and enhance the half-life of the phytochemicals (206, 207).

Chitosan capped biogenically synthesized silver nanoparticles containing vincristine prepared by varadharajan et al. showed significant cytotoxicity against A549 lung cancer cells in a dose-dependent manner. The acidic extracellular pH exhibited by the tumor cells further helped in specific targeting, and the nanoparticle showed an IC 50 value of 1.033 μg/ml against A549 human lung cancer cells (208). Li et al. formulated N-acetyl-L-cysteine capped ZnO nanoparticles containing Camptothecin (CPT), a natural antitumor alkaloid isolated from the Chinese tree *Camptotheca acuminate.* The hemolytic assay revealed the absence of any hemolytic activity by the fabricated ZnO nanoparticle. Further, the Cytotoxicity assessment of the metal nanoparticle on human A549 cell lines showed a drop in IC50 values from 1.17 μg/mL (free CPT) to 0.66 μg/mL (ZnO-NAC-CPT NPs). The results of the study augment the potential of the prepared N-acetyl-L-cysteine capped ZnO nanoparticles containing Camptothecin (CPT) against lung cancer (209). Thidinjam et al. fabricated iron oxide nanoparticles were containing *Phyllanthus emblica* fruit extract for *in vitro* testing against A549 human lung cancer cell lines. The Phytochemical tests carried out on the extracts revealed the presence of alkaloids. The prepared iron oxide nanoparticles increase reactive oxygen species production, and cause more severe DNA damage and apoptosis in the A549 human lung cancer cell line at a 100µg/ml concentration (210). Another study demonstrated the anti-carcinogenic effect of gold nanoparticles prepared using ethanolic extract of *Plectranthus amboinicus* against human LAC cells (A549) *in vitro*. About 50% cell death was observed at 80 μg/mL of Au NPs exposed to A549 cells. The toxicity of the gold nanoparticles also varied according to the shape and size, those with a size <2nm were found to be more toxic than larger sizes (211).

Recent work by Zhang H et al. showed that Zinc oxide nanoparticles containing roots of *Euphorbia fischeriana* exhibited anti-cancer activity against lung cancer cell lines by modulation of apoptotic signaling pathways. The phytochemical tests revealed the presence of alkaloids in the aqueous root extract of *Euphorbia fischeriana*. The prepared Zinc oxide nanoparticle also exhibited cytotoxicity against human lung cancer cells at a concentration range of 14.5 µg/ml. The *Euphorbia fischeriana* Zinc oxide (EF-ZnO) nanoparticles further inhibited cell migration and induced pro-apoptosis, exhibiting anti-cancer potential (212).

SnO2 Nanoparticles using *Piper nigrum* were synthesized by Tammina et al. and evaluated for cytotoxicity against A549 lung cancer cell lines. It was observed that SnO2 Nanoparticles containing *Piper nigrum* extracts had IC_50_ values of 135, 157 and 187µg/ml, respectively, against A549 lung cancer cell lines. In conclusion, The SnO2 NPs synthesized by a cost-effective and non-hazardous green synthetic route is an effective alternative to currently available lung cancer therapies (213).

Recently Fahmy et al. formulated platinum and palladium nanoparticles using *Peganum harmala* seed alkaloid fraction. The nanoparticles mixture demonstrated significant antioxidant activity of 843.0 ± 60 µM. Furthermore, the metallic nanoparticles exhibited substantial anti-cancer activity against A549 cell lines with IC50 values of 8.8 µg/ml (214).

#### 3.4.2 Magnetic nanoparticles (MNPs)

MNPs are a significant class of nanomaterials that can transform existing cancer therapeutics. The magnetic nanoparticles generally lie in the size range of 1-100nm, and an external magnetic field controls the release of the active moiety from them. These magnetic-based drug delivery systems can successfully deliver a concentrated amount of drug to the target tissue, thereby increasing the therapeutic activity, bioavailability and reducing dose frequency (215, 216).

Magnetic nanoparticles have demonstrated their promising potential in treating lung cancer because of their hypothermic effect and active targeting abilities (217).

Castillo et al. developed iron oxide superparamagnetic nanoparticles were containing Camptothecin (CPT), a quinoline based alkaloid. CPT has not been used as a chemotherapeutic agent due to stability and solubility aspects. When Camptothecin has loaded into iron oxide superparamagnetic nanoparticles with the help of polyethylene glycol, it demonstrated remarkable pro-apoptotic activity against H460 lung cancer cell lines (218).

In another similar study, Zhang et al. fabricated Hydroxycamptothecin-loaded Fe3O4 nanoparticles and tested their anti-cancer potential using human lung cancer cell line HCC827. The Hydroxycamptothecin-loaded nanoparticles demonstrated their proliferative effect by reducing the tight junction function and activating the caspase-8 pathway with increased pro-apoptotic activity (219).

**Figure 2:**
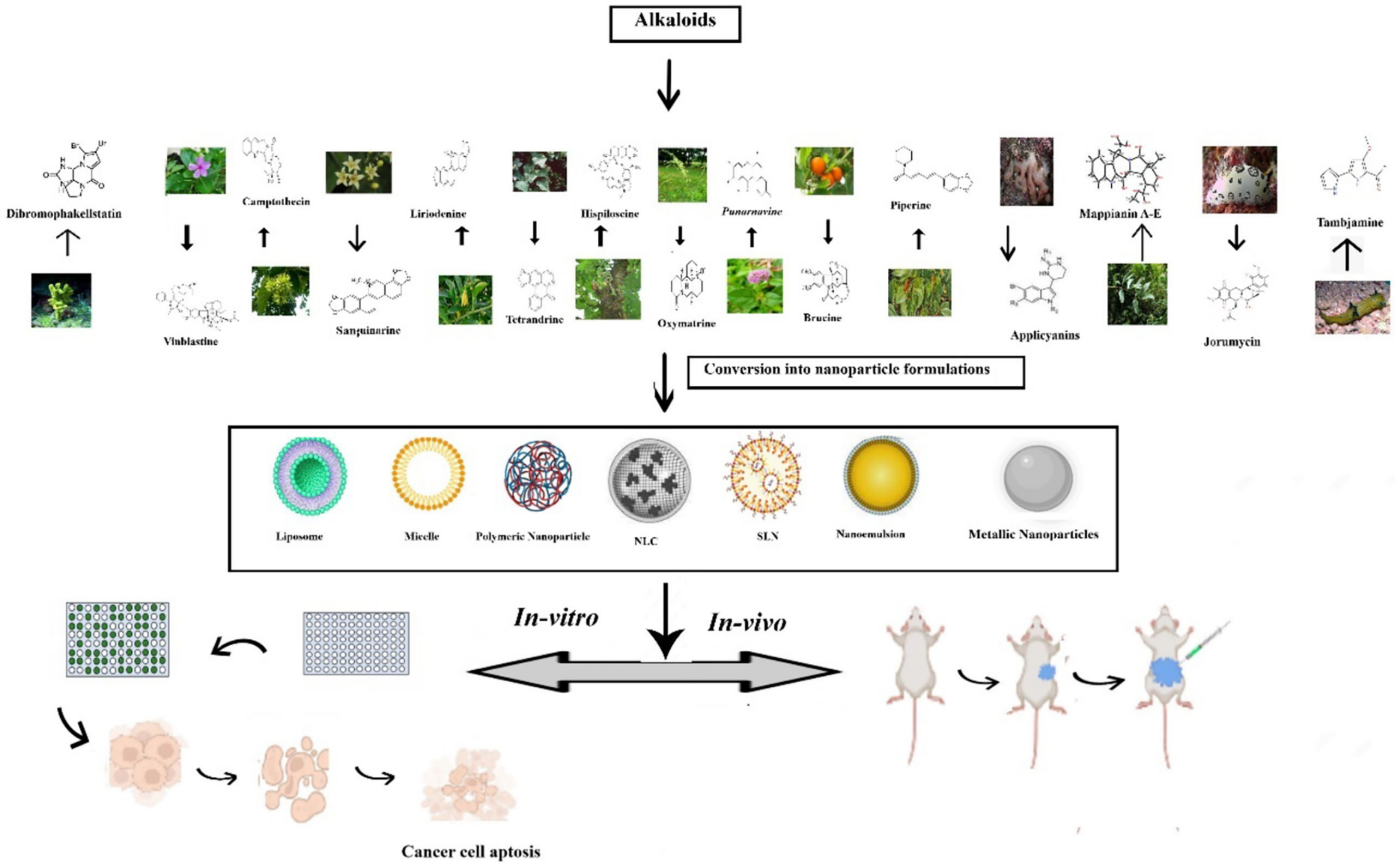
Nanoformulations of alkaloids in the management of lung cancer.

Wang et al. prepared and evaluated the anti-cancer effect of magnetic Fe3O4-loaded poly(lactic-co-glycolic) nanoparticles of Tetrandrine (Tet), a bisbenzylisoquinoline (BBI) alkaloid. The superparamagnetic iron oxide (Fe3O4) nanoparticles containing the natural drug tetrandrine could easily permeate the A549 cell lines and exhibit cytotoxicity, demonstrating their anti-proliferative potential. Furthermore, the western blot studies revealed that the anti-proliferative effect was achieved by damaging the lysosomes and thus activating the mitochondrial pathway and thus inducing a549 cell apoptosis (220). A-List of some vital alkaloid nanoformulations employed in lung cancer therapy is given in **Table 4**. **Figure 2** shows the nanoformulations of alkaloids in the treatment of lung cancer.

**Table 4:** Alkaloid nanoformulations for treatment of lung cancer.

Alkaloidal anticancer drug	Nanoformulation type	Type of study	Model used	Reference
Docetaxel	Liposome	*In vivo*	A549 xenograft rat	(221)
		*In vitro*	A549 cell lines	(222)
		*In vitro*	A549 cell lines	(223)
	Micelle	*In vitro*	A549 cell lines	(224)
	Nanostructured lipid carrier	*In vitro*	A549 cell lines	(225)
	Nano-extraction	*In vitro*	A549 cell lines	(226)
Paclitaxel	Liposome	*In vivo*	A549 xenograft mice	(227)
		*In vitro*	A549 cell lines	(228)
		*In vivo*	Mice, mouse	(229)
	Polymeric micelles	*In vivo*	Rats	(230)
	SLN	*In vivo*	Mice	(231)
		*In vitro*	MXT-B2 cell lines	(231)
Topotecan	Liposome	*In vitro*	LLC	(232)
Berberine	SLN	*In vitro*	A549 cell lines	(233)
Evodiamine of fructus bruceae oil	Nanoemulsions	*In vivo*	Rats and mice	(234)
Vinblastine	Cationic liposome	*In vitro*	LLT cells	(235)
	Cationic liposome	*In vivo*	C57BL/6 mice	(235)
Irinotecan	Liposome	*In vivo*	Xenograft mouse	(236)
9-nitro camptothecin	Liposome	*In vivo*	Mice	(237)
β-carboline and quinazoline alkaloids	Platinum and Palladium Nanoparticles	*In vitro*	A549 cell lines	(238)
Alkaloid extract from *Derris trifoliata*	Silver nanoparticles	*In vitro*	A549 cell lines	(239)
Alkaloid extract from *Justicia adhatoda*	Gold nanoparticles	*In vitro*	A549 cell lines	(240)
Alkaloid extract from *Indigofera tinctoria*	Silver and gold nanoparticles	*In vitro*	A549 cell lines	(241).

## 4 Scientific prospection

**Figure 3:**
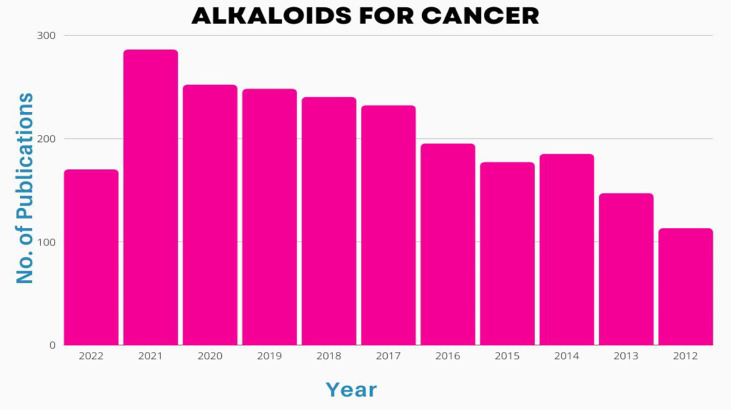
Number of publications [2002-2022] on basis of alkaloid application in treatment of cancers.

**Figure 4:**
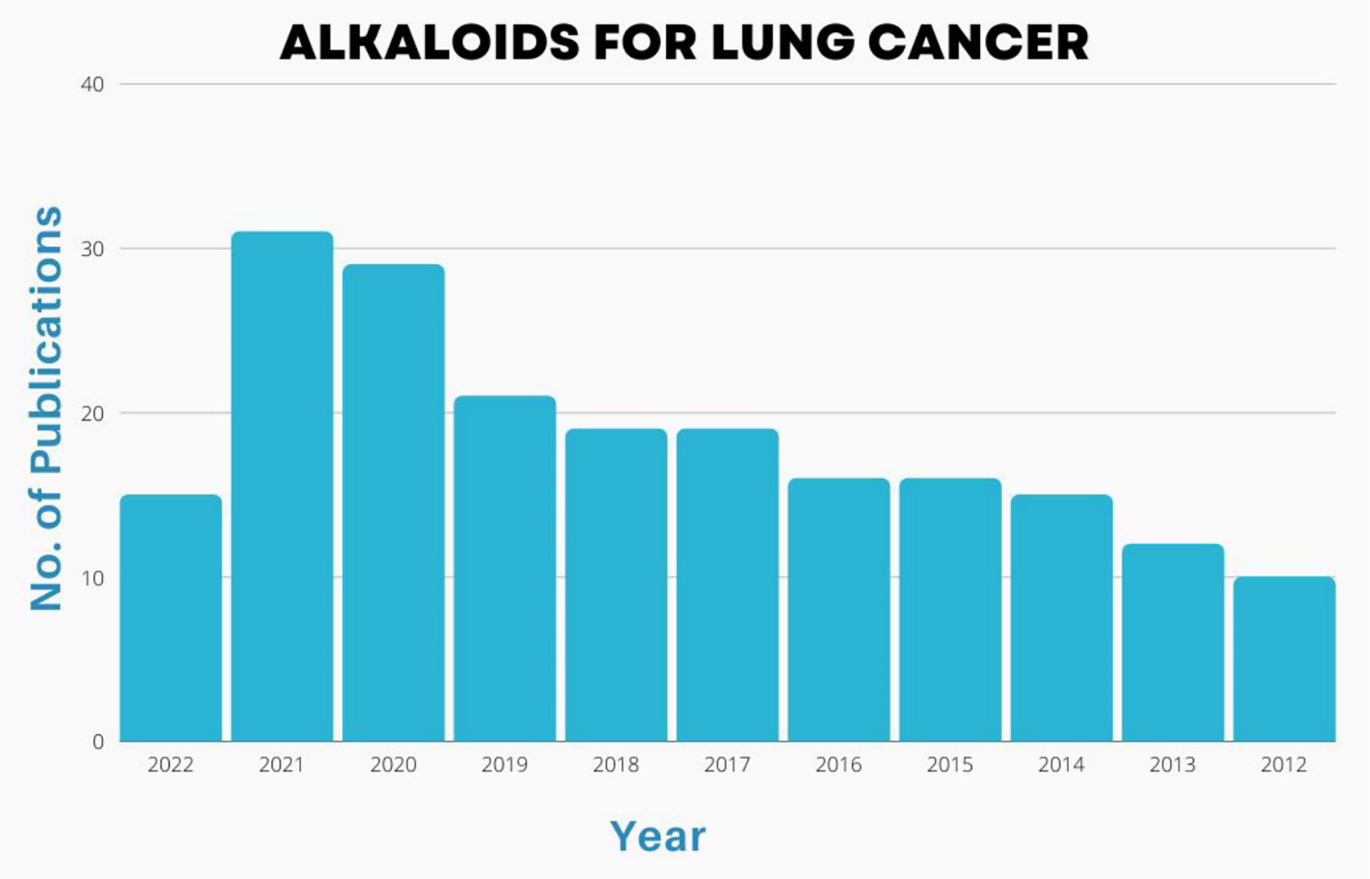
Number of publications [2002-2022] on basis of alkaloid application in treatment of lung cancers.

Alkaloids are important chemical compounds that serve as a rich source for drug discovery. Numerous alkaloids screened from medicinal plants and herbs showed antiproliferative and anticancer effects on wide category of cancers both *in vitro* and *in vivo*. A scientific prospection was made from 2012 to 2022 using PubMed database by using two search queries i) “(alkaloid[Title/Abstract]) AND (cancer[Title/Abstract])” and ii) (alkaloid[Title/Abstract]) AND (lung cancer[Title/Abstract]) and results were represented in **Figures 3**, **4**. **A** total of 2245 publications on alkaloids for lung cancer were resulted. The same was filtered for application of alkaloids for treatment of lung cancer and the results were summarized in 203 publications. Starting with 2012, there were only 10 publications focused on this. But the numbers have been drastically increased since afterwards. The number of publications reached to maximum of 31 in 2021. In 2022, already witnessed 15 manuscripts and the number will cross 35-40 by the end of the year. Similar trend was observed for alkaloids for the treatment of all types of cancers. The total number of manuscripts on this content in 2012 is only 113. Within 5years, there is a strong shoot up of publications with 232; 252 in 2020; 286 in 2021 and 170 in till date of 2022. Based on the scientific prospection, one can confirm that the alkaloids represent an important group of anticancer drugs of plant origin with enormous potential for future development of drugs for cancer therapy and management. The enormous growth in research of this area can be evident from this prospection.

Liposomes efficiently encapsulated curcumin and bromocriptine (BR) in a polymer structure, which results in enhanced aqueous solubility of the mentioned hydrophobic agents and higher bioavailability of the drugs. Preparation of curcumin and BR liposomes were carried out by the thin film method, and the amounts of purified drug and its release were analyzed. After dose determination, the human lung cancer cells (QU-DB) were exposed to BR and curcumin liposomes for 12, 24, and 48 h. Then the viability and apoptosis assays were carried out by using tetrazolium dye and flow cytometry technique, respectively. *In vitro* anti-cancer effects of former nano-formulations on lung cancer cells was confirmed, and no cytotoxicity effects of these nano-preparations were observed in the normal cells (HFLF-PI5).

Curcumin and bromocriptine (BR) were successfully encapsulated in liposomes using a polymer framework, increasing the medications’ bioavailability and improving their solubility in water. The levels of pure medication and its release were assessed after the thin film method was used to prepare curcumin and BR liposomes. The human lung cancer cells (QU-DB) were treated to BR and curcumin liposomes for 12, 24, and 48 hours following dose determination. Then, using tetrazolium dye and the flow cytometry technique, the viability and apoptosis experiments were performed. Former nano-formulations’ *in vitro* anti-cancer activities on lung cancer cells were verified, and no cytotoxicity effects of these nano-preparations were seen in normal cells (HFLF-PI5).

The new bisbenzylisoquinoline alkaloid, known as bersavine, was discovered in the Berberis vulgaris L. (Berberidaceae) plant. A 48-hour cytotoxicity study revealed that bersavine significantly reduces the viability and proliferation of cancer cells from the cervix (HeLa), colon (HT-29), breast (MCF-7) and leukemia (Jurkat, MOLT-4), with IC50 values ranging from 8.1 to 11 M. Leukemic Jurkat and MOLT-4 cells’ survival and proliferation were reduced following bersavine treatment in a time- and dose-dependent manner. Using an xCELLigence assay, bersavine demonstrated concentration-dependent antiproliferative efficacy in human lung, breast, ovarian, and hepatocellular cancer cell lines. Using the flow cytometry technique, significantly more MOLT-4 cells were arrested in the G1 phase of the cell cycle after being exposed to bersavine at a concentration of 20 M for 24 hours. After 24 hours of bersavine administration, a greater percentage of apoptotic cells was detected. The advancement of MOLT-4 cell death was accompanied by the overexpression of p53, which was phosphorylated on Ser392. In human leukemic cells, lower proliferation appears to depend on increased Chk1 Ser345 phosphorylation and decreased Rb Ser807/811 phosphorylation, whereas bersavine-induced death is a result of enhanced caspase activity.

Investigating the *in vitro* antiproliferative and antimigratory effects of berberine-loaded liquid crystalline nanoparticles (LCNs) in a human lung epithelial cancer cell line (A549). The created nanoparticles were discovered to have sustained release behavior, high entrapment effectiveness, and an average particle size of 181.3 nm with spherical shape. The considerable reduction of proliferation, colony formation, invasion or migration *via* epithelial mesenchymal transition, and proliferation-related proteins linked to cancer progression were the most notable results reported with berberine-loaded LCNs. According to the research, anti-cancer drugs with low solubility and bioavailability can be improved upon by being formulated into delivery systems based on nanotechnology. With different molar ratios of the components, berberine was noncovalently linked to the carbon nanostructure C60 fullerene (C60) and tested against Lewis lung cancer (LLC). With medium acidity, ber release from C60-Ber nanocomplexes was accelerated. Treatment of LLC cells with C60-Ber nanocomplexes led to increased intracellular absorption of Ber relative to free Ber. At the conclusion of the experiment, the 2:1 C60-Ber nanocomplex group experienced a 50% reduction in tumor growth, but the tumor-bearing group treated with free Ber showed no therapeutic benefit (14, 242).

## 5 FDA associated information

An examination of all FDA-approved small-molecule drugs from 1981 to 2014 found that roughly 51% were natural compounds and their derivatives, and that this percentage rose to about 80% for anti-cancer small-molecule drugs. Natural products have been categorized into a number of classes, including terpenoid, polyketide, phenylpropanoid, and alkaloid. Numerous alkaloids have been successfully identified and used for the benefit of mankind since the first alkaloid, morphine, was commercialized in 1826. The FDA has now approved alkaloid medications for the treatment of a variety of conditions, including cancer, Alzheimer’s disease, Parkinson’s disease, migraines, pain management, erectile dysfunction, heart failure, and more. The most recent example of this application of nanotechnology is VYXEOS, which may deliver numerous medicinal compounds in co-loaded nanoparticles. Acute myeloid leukemia can be treated with VYXEOS, a liposomal formulation of daunorubicin and cytarabine with a synergistic molar ratio of 1:5.

The therapy of Philadelphia chromosome-negative acute lymphoblastic leukemia with liposomal vincristine (Marqibo), which was licensed by the FDA in 2012, was the first anticancer alkaloid to be successfully encapsulated in lipid-based nanoparticles. Vincristine’s dosing restrictions were overcome by Marqibo in the phase I trial that resulted in the FDA’s approval of the drug. This allowed vincristine to be administered at the dose of 2.25 mg/m2 without dose capping or apparent toxicity worsening. Additionally, Marqibo has better pharmacokinetic characteristics than free vincristine, showing extended plasma circulation, a lower clearance, and a greater AUC. The approved adult weekly dose of 2.25 mg/m2 was well tolerated and exhibited no signs of neurotoxicity, according to a phase I trial done in children. In 2015, the FDA approved liposomal irinotecan (Onivyde) for the treatment of metastatic pancreatic ductal adenocarcinoma (mPDAC) in patients who had previously received gemcitabine-based therapy in combination with 5-fluorouracil and folinic acid.

## 6 Future prospective

In the management of lung cancer, phytoactive compounds like alkaloids have attracted great interest due to their peculiar features. In addition, to improve bioavailability, drug efficacy has been significantly enhanced through the controlled drug release of these nanocarriers. In lung cancer treatment, it is expected that nano-based drug delivery systems will improve and unlock a new dimension. Extensive research in this area is expected to try new models and replace traditional delivery methods with novel drug delivery systems, shortly leading to better health care delivery systems. Developments in this area have already helped address non-specific targeting issues, low therapeutic potential, unintended side effects, and traditional medical interventions such as drug resistance. These nano-based drug delivery systems have become ideal carriers for lung cancer. Liposomes, solid lipid nanoparticles, nanostructured lipid carriers, polymeric nanoparticles, magnetic nanoparticles, and many more have been developed and for targeting the infected sites; lipid-based nanoparticles, especially liposomes, have been researched and exploited in a wide range of products due to their distinctive and attractive properties for drug distribution. Therefore, encapsulation of lipid-based nanoparticles has proven to be a powerful mechanism for advancing the clinical translation of anticancer alkaloids in lung cancer treatment of lung cancer. Nanoparticles with multiple structures allow surface changes to deliver water-insoluble drugs and the ability to cross biological barriers and target the desired site in the body. This therapeutic approach has raised hopes in patients with lung cancer and has received an excellent response due to its potential for site-specific targeting with low toxicity. However, currently used anticancer alkaloids in clinical practice are limited by the lack of specificity of cancer cells, the unselective tissue distribution, and the severe side effects caused by toxic formulation excipients. Nanotechnology provides access to a broad molecular toolbox that may be altered for application in respiratory oncology due to the distinct physicochemical features of materials. This realization has led to the approval of several NP formulations in clinical studies, as well as the development of many more.

Due to advancements made possible by nanotechnology, the number of treatments for NSCLC is growing. Because of the addition of nanomedicine to conventional medicines, their toxicity and clinical results have improved. Drugs are concentrated in the right cell populations using nanodelivery devices, which also control drug release to enhance long-term benefits. Furthermore, early trials of newly developed immunotherapies for the treatment of cancer are promising. The most promising cancer methods involve new therapeutics based on siRNA, mRNA, and gene editing. The seamless fusion of these therapies with the adaptability of nanotechnology and their concurrent development indicate that the effects of these treatments on patient lives will become apparent in the not too distant future. However, nanotechnology still needs to advance. It is challenging to fully comprehend the underlying mechanisms of the interactions between nanoparticles and biomolecules and, as a result, to enhance treatment planning. Furthermore, as the present EPR paradigm is being seriously questioned, research is needed to explain the phenomena of tumor nanoparticle permeability.

## 7 Conclusion

Around 65-70% of the anticancer drugs introduced over the last two decades have been acquired from natural sources. Among compounds obtained from natural sources, Plant-derived alkaloids have emerged as potential candidates in lung cancer therapy. However, Phyto alkaloids ache for solubility and poor bioavailability. Applying nanotechnology to these natural compounds enhances bioavailability, prolongs drug circulation, improves therapeutic efficacy, and reduces toxicity. From the current manuscript, it is pronounced that there is exhaustive availability of alkaloids, which could be explored in various nanoformulations for their practical application in treating several lung cancers. Different types of alkaloids and their nanoformulations for the management of lung cancer were discussed elaborately. Nevertheless, there are gaps and research initiatives to formulate several tumor-targeted nanotherapeutic systems with the desired yield and quality of alkaloids obtained from natural sources. Based on the provided information, we believe several researchers will soon initiate nano delivery of alkaloids that will work effectively on the specific type of lung cancer. The authors conclude with a precautionary note that all the alkaloid nanoformulations discussed in the review are based on *in vitro* or animal-based models of lung cancer. Further extension of these studies in healthy and cancerous subjects is necessary for their potential commercialization.

## Author contributions

SS and NN conceptualized and designed the manuscript, participating in drafting the article and/or acquisition of data, and/or analysis and interpretation of data. GR, GG, HC, MP, MA, and JJ prepared the figures and tables. JJ, TE, and BK wrote, edited and revised the manuscript critically. JJ, TE, and BK revised the final written. All authors critically revised the manuscript concerning intellectual content and approved the final manuscript.

## Funding

This research was supported by Basic Science Research Program through the National Research Foundation of Korea (NRF) funded by the Ministry of Education (NRF-2020R1I1A2066868), the National Research Foundation of Korea (NRF) grant funded by the Korea government (MSIT) (No. 2020R1A5A2019413), a grant of the Korea Health Technology R&D Project through the Korea Health Industry Development Institute (KHIDI), funded by the Ministry of Health & Welfare, Republic of Korea (grant number: HF20C0116), and a grant of the Korea Health Technology R&D Project through the Korea Health Industry Development Institute (KHIDI), funded by the Ministry of Health & Welfare, Republic of Korea (grant number: HF20C0038).

## Conflict of interest

The authors declare that the research was conducted in the absence of any commercial or financial relationships that could be construed as a potential conflict of interest.

## Publisher’s note

All claims expressed in this article are solely those of the authors and do not necessarily represent those of their affiliated organizations, or those of the publisher, the editors and the reviewers. Any product that may be evaluated in this article, or claim that may be made by its manufacturer, is not guaranteed or endorsed by the publisher.
